# Soil Oxidation-Reduction in Wetlands and Its Impact on Plant Functioning 

**DOI:** 10.3390/biology1020196

**Published:** 2012-07-26

**Authors:** S. R. Pezeshki, R. D. DeLaune

**Affiliations:** 1 Department of Biological Sciences, University of Memphis, Memphis, TN 38152, USA; 2 Department of Oceanography of Coastal Sciences, School of Coast & Environment, Louisiana State University, Baton Rouge, LA 70803, USA; Email: rdelaune@aol.com

**Keywords:** flooding, photosynthesis, plant stress, soil redox potential, soil phytotoxins, wetlands

## Abstract

Soil flooding in wetlands is accompanied by changes in soil physical and chemical characteristics. These changes include the lowering of soil redox potential (Eh) leading to increasing demand for oxygen within the soil profile as well as production of soil phytotoxins that are by-products of soil reduction and thus, imposing potentially severe stress on plant roots. Various methods are utilized for quantifying plant responses to reducing soil conditions that include measurement of radial oxygen transport, plant enzymatic responses, and assessment of anatomical/morphological changes. However, the chemical properties and reducing nature of soil environment in which plant roots are grown, including oxygen demand, and other associated processes that occur in wetland soils, pose a challenge to evaluation and comparison of plant responses that are reported in the literature. This review emphasizes soil-plant interactions in wetlands, drawing attention to the importance of quantifying the intensity and capacity of soil reduction for proper evaluation of wetland plant responses, particularly at the process and whole-plant levels. Furthermore, while root oxygen-deficiency may partially account for plant stress responses, the importance of soil phytotoxins, produced as by-products of low soil Eh conditions, is discussed and the need for development of methods to allow differentiation of plant responses to reduced or anaerobic soil conditions *vs*. soil phytotoxins is emphasized.

## 1. Introduction

Excess water in wetland soils is a major factor affecting plant survival and functioning. In saturated soils, the supply of atmospheric oxygen into the soil is curtailed and various facultative and obligate anaerobic microorganisms use oxidized compounds as electron acceptors for respiration, thus converting them to reduced forms. The reduction and the associated processes influence plant survival, growth and functioning in wetlands. This review focuses on the literature relevant to this topic and the need for development of research methods associated with properly quantifying of soil reduction processes that can aid in the evaluation of plant responses in wetland environments. 

## 2. Soil Oxidation-Reduction

A chain of reactions is initiated upon soil flooding leading to reduced (low) soil redox potential (Eh, mV) conditions. These reactions include physical, chemical and biological processes that have significant implications for wetland plants [1,2,3]. Physical processes include restriction of atmospheric gas diffusion in the soil leading to depletion of soil oxygen and accumulation of carbon dioxide [4,5]. Shortly after flooding, the limited supply of oxygen in soil pore spaces is depleted rapidly by roots, microorganisms, and soil reductants [6]. This process leads to oxygen depletion and reduction in soil oxidation reduction potential (Eh) followed by a chain of soil chemical changes. The processes that follow include denitrification, reduction of iron, manganese and sulfate, and changing soil pH and Eh [3]. For example, in a typical series of reductions NO_3_^−^ is reduced to N2, Mn^+4^ to Mn^+2^, Fe^+3^ to Fe^+2^, SO_4_^2−^ to H_2_S, S^2+^ or HS^−^ (depending upon pH) and accumulations of acetic and butyric acids that are produced by microbial metabolism [1,2]. 

## 3. Quantifying Soil Redox Potential Conditions

Review of literature reveals that many terms such as “flooded”, “saturated”, “waterlogged” are utilized to describe oxygen-deficient root medium. Obviously, these terms are qualitative and do not quantitatively define the rhizosphere [7]. Furthermore, methods used to quantify oxygen content and oxygen diffusion rate in drained soils cannot be employed effectively in wet soils [3]. In contrast, measurement of soil Eh is a valuable tool because it can be measured in laboratory and in the field (for details see [8,9] and the references cited therein). Furthermore, quantifying soil Eh is particularly advantageous in periodically flooded soils since the range of Eh is much wider, between approximately −300 to +700 mV, than either aerated (Eh > +400 mV) or permanently waterlogged (Eh < +350 mV) soils [10,11,12], Thus, soil Eh or redox measurements represent an excellent quantifying tool for defining the presence or absence of oxygen and the soil chemical status in wetland soils. 

## 4. Intensity and Capacity of Soil Reduction

**Figure 1:**
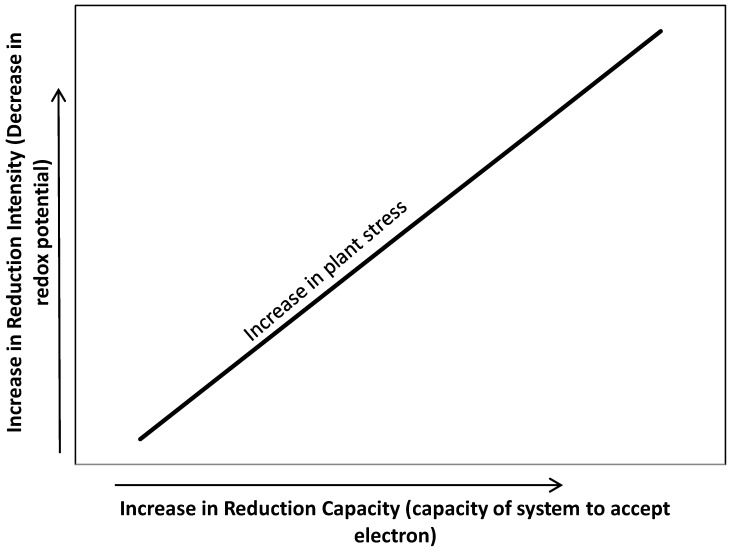
Oxidation-reduction potential in wetland soils: relationship of intensity and capacity of reduction in soil to wetland plant stress.

In wetland soils, the oxidation-reduction is used to signify the intensity of reduction. However, the reduction of inorganic redox systems including oxygen following flooding can be described in “intensity” and “capacity” terms [7]. Although critical Eh values (intensity of reduction) at which the inorganic redox systems become unstable do provide valuable information, they do not provide any indication of the total capacity of the system to accept electrons, supporting respiration or oxygen demand in the root zone [9]. In contrast, redox capacity represents the amount of electrons accepted by oxidants (such as oxygen, nitrate, manganese, iron, and sulfate) supporting respiratory of microorganism decomposing organic matter. The oxidants may have a relative high redox buffering capacity which explains why some wetland soil with relatively large amount of bioreducible iron do not undergo a rapid decrease in redox potential (intensity) following flooding. High soil reduction capacity at any intensity of reduction in the root zone can compete for oxygen transported thru aerenchyma into the root rhizosphere and potentially can increase plant stress above that caused by intensity of reduction alone (Figure 1).

The intensity factor determines the relative ease of the reduction, while the capacity factor denotes the amount of redox system undergoing reduction, e.g., oxygen consumption at root interface [7,13,14]. This relationship may be expressed as follows: Eh = Eo + 2.3 [RT/nF] log_e_ [Ox/Red]; where Ox and Red are the concentrations of the oxidized and reduced forms, respectively, of the substance under consideration, Eo is the electrode potential of the 50% oxidized system specific to that substance, n is the number of moles of electrons transferred, F is the Faraday constant, R is the gas constant, and T is the temperature (K) [12]. It then follows that Eh is dependent on the ratio of oxidized and reduced forms and not on their absolute quantities. Therefore, a 90% oxidized system has the same electrode potential no matter whether the total concentration is 0.01% or 10%, but the poising (capacity) of the latter will be 1000 times greater. Thus, a system of Eo + 0.1 volt will oxidize a system of Eo − 0.1 volt, but will be oxidized by a system of Eo + 0.3 volts; however, the extent to which the reactions will take place depend upon the capacity of the systems.

The redox capacity factor is important although less is known about whether it is the intensity or the capacity that is most affecting plant functioning. As noted above, two soils with the same level of intensity of reduction may differ substantially in the capacity for reduction. The reduction capacity is estimated using measurements of soil respiration and calculating oxygen equivalent by stoichiometry [14]. However, part of the oxygen consumption is due to microbial respiration and partly due to direct oxidation of the accumulated reductants. Development of methodology for distinction of these two components therefore, is needed. In the laboratory, levels of soil redox capacity may be created and/or manipulated by adding extra carbon (organic matter) as energy source to the soil while maintaining the redox intensity level. In an experimental set up, reduction capacity may be controlled by adding granular D-glucose to the growth medium that is maintained under set redox intensity conditions [15].

To properly evaluate wetland plant responses to soil flooding, both intensity and capacity of soil reduction must be quantified because these two components influence oxygen demand in the soil [7,14]. Kludze *et al*. [16,17] and Sorrell *et al*. [18] using titanium citrate solution to create a high O_2_-demand in root environment, reported that root oxygen transport and release were affected by such conditions in several wetland species. However, such a solution, while a significant improvement over de-oxygenated solution, at best only mimics wet soil conditions [14] and does not represent other important characteristics of wet soils, such as soil capacity for phytotoxin production that may have significant adverse effects on many wetland plants. 

## 5. Soil Reduction and Wetland Plant Functioning

Responses of plants to low oxygen in the root zone have been typically assessed by growing plants hydroponically, then introducing pressurized nitrogen through the solution to remove oxygen [7]. In such a system, roots are exposed to Eh only slightly below values where oxygen disappears on the redox scale (*i.e.*, +350 to +400 mV; [7]). However, low oxygen conditions are represented by Eh values ranging between +400 and −300 mV. The Eh around −300 mV may occur in highly reduced soils. Since oxygen is absent at Eh values beginning at or below +350 mV, the absence of oxygen alone does not provide much information on the intensity of reduction. Furthermore, studies designed to evaluate responses of plants grown at the upper portion of the anaerobic range of the redox scale may yield results that are not typically the same as those displayed by plants growing in a more reducing environments.

Based on the considerations outlined above, it is argued that the common laboratory approaches of pressurized N_2_ may be sufficient for evaluation of responses of flood-sensitive plants, such as many crop species, to low oxygen conditions. However, evaluation of responses of wetland plants to oxygen-deficient soils require methods that represent root zone reduction at levels at which flood-tolerant wetland species are subjected to in natural wetland soil enviornment. These plants can survive low to extreme reducing conditions covering a significant portion along the redox scale in their natural environments.

Clearly, in wetland soils, plants are faced with not only the lack of oxygen but a substantial demand for oxygen in the sediment due to plant roots and microbial demand [7,9,13,19,20,21,22,23,24,25,26]) that is represented by low soil Eh. Extensive field data on the relationship between Eh and plant community distribution in saltmarshes were presented by Armstrong *et al*. [27]. Pennington and Walters [28] and Davy *et al*. [29] reported sediment Eh as a major factor affecting survival and growth of their study species. It is known that in sediments characterized by weak redox capacity, certain wetland plants are capable of increasing the Eh of the bulk sediment [30,31,32]. 

In a typical flooded soil, plants may respond to soil physicochemical changes by displaying a wide range of stress symptoms that have been reported in numerous publications (for reviews see [19,22,23,33,34,35,36]. However, due to lack of quantifying Eh conditions in many reports, the relationship between soil Eh and wetland plant functioning is less understood. From a physiological-ecology standpoint, soil Eh data provide insights into the status of various soil compounds, many of them important to wetland plant functioning. For example, a soil Eh of zero mV indicates that oxygen and nitrate are not likely to be present while the bioreducible iron and manganese compounds are in a reduced state. Eh reading of +400 mV indicates that oxygen may be present despite the presence of excess water in the soil [7,37,38].

A major consequence of soil flooding and the subsequent reducing conditions is development of high competitive oxygen between root and soil microbial demand that may affect internal plant tissue oxygen concentrations thus, its critical metabolic processes [7,19,21,26,39,40,41]. Other consequences of soil reduction processes include changes in availability and/or concentrations of various nutrients that are essential for plant functioning, and production of a host of compounds known to be phytotoxic [3,8,42,43]. These compounds include: reduced forms of Fe and Mn, ethanol, lactic acid, acetaldehyde and aliphatic acids such as formic, acetic, butyric acids, and cyanogenic compounds [2,6,44]. The accumulation of these compounds in flooded soils may reach levels that can cause injury to plants [1,9,45]. For instance, excess soil sulfide is known to inhibit growth of various marsh macrophytes [20,21,46,47,48]. The soluble sulfide species including H_2_S can be toxic to plant roots [38,49,50]. The organic acids such as acetic, butyric, propionic and caproic acids have a variety of adverse effects on plants [20,21,38,51,52]. 

In the following sections, the effects of soil flooding, and the concomitant drop in soil Eh, on certain plant metabolic processes, physiological functions, anatomical and morphological features, growth and survival is discussed. 

## 6. Metabolic Responses to Flooding

**Figure 2:**
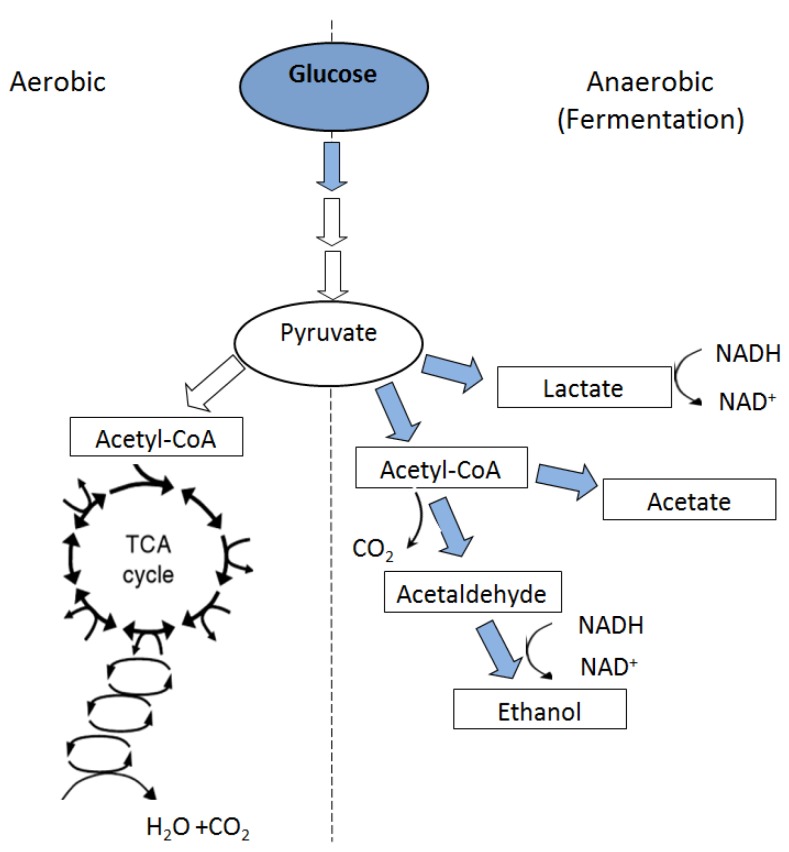
Metabolic pathways under aerobic and anaerobic conditions (from Reddy and DeLaune [9]).

Metabolic adaptations are complex mechanisms that allow wetland plants to survive anoxic soil conditions [8,38]. Normal growth and functioning of roots require more oxygen than is needed for root respiration processes alone [4,53]. Under aerated conditions, oxygen primarily diffuses into the roots from soil air spaces via the root epidermis. However, when roots are flooded, the presence of water column in soil creates a severe barrier to gas diffusion, thus oxygen must reach the roots through internal paths from aerial parts [38,54]. Such conditions lead to root energy deficiency because of the inhibition of normal (aerobic) root respiration. Therefore, much of the immediate flood-injury to roots is attributed to their oxygen status [4,28,55]. Under root oxygen stress, mitochondrial respiration is compromised while metabolism is shifted to fermentation. The process converts pyruvate to ethanol (Figure 2) via activity of enzyme alcohol dehydrogenase (ADH) or to lactate via activity of lactate dehydrogenase (LDH) [56]. 

Increased ADH activity in response to flooding has been reported for many plants as an indicator of oxygen deficiency [57,58,59]. The response has been reported for flood-sensitive as well as flood-tolerant plants [60,61]. The role of ADH in flood-tolerance has been known for sometime and may include maintaining intracellular pH. The ability to survive is related to the extent of cytosolic acidosis due to accumulation of organic acids from fermentation, such as lactate. The changes in cell pH have considerable impact on cell metabolism due to reduction in activities of several enzymes that require pH close to 7 for optimum activity [62]. The ADH activity appears to be inversely related to soil Eh due to anaerobic respiration [9]. Nonetheless, it is not known if the induction of ADH activity contributes to the plant survival or signals the harmful effects of root anoxia [54].

Major products of fermentation are ethanol and CO_2_. Anaerobic respiration, however, is rather an inefficient pathway of energy production because it produces 2 moles of ATP per each glucose molecule broken down to pyruvic acid compared to 36–38 ATPs generated from each glucose in aerobic respiration. The low ATP production creates an energy deficiency that affect many plant metabolic functions including water and nutrient uptake, internal solute transport, and photosynthetic carbon fixation. Additionally, the increased glucose consumption during fermentation may lead to carbohydrate depletion in plants leading to low growth. The metabolic processes affected may include reduction in activity of photosynthetic enzymes. The activity of these enzymes is highly sensitive to changes in environmental conditions [63,64,65,66]. In addition, decrease in leaf tissue chlorophyll content has been reported in response to environmental stresses including soil flooding [66,67]. Many species rely on anaerobic metabolism as a means of surviving anaerobic root conditions yielding very few molecules of ATP [4,68]. Wetland plants maintain carbohydrate reserves to support sustained ATP generation [69,70]. Thus, the carbohydrate stored in the roots may potentially play an important role in plant survival since presumably the carbohydrates can be used to feed anaerobic respiration under reduced soil Eh conditions. 

The ability to survive flooding is also related to avoidance of ethanol production in glycolytic pathway. Ethanol accumulation in flood-sensitive species may lead to death of root cells, therefore, to avoid excess accumulation of ethanol, it may be leaked out of plant via transpiration stream and/or leakage as well as production of alternate end products such as malate and lactate. Reduction of water and nutrient uptake, disturbance of hormonal balance such as a decrease in gibberellin and cytokinin and an increase in abscisic acid and ethylene [71] due to flooding has been reported in the literature [72,73].

## 7. The Internal Oxygen Transport System

Root and rhizomes in flooded wetland plants obtain oxygen via gas-phase transport from the shoot system, internal photosynthetic oxygen production, or atmospheric oxygen through an extensive oxygen transport system (aerenchyma tissue) in roots, stems, and leaves [17]. This system allows a plant to transport the needed oxygen to the roots for maintaining aerobic respiration and to oxidize reducing compounds in the rhizosphere, thus establishing a gradient of soil oxygen availability. The role that roots of certain wetland woody species have on establishing such radial gradients in a soil profile that influences soil redox status has been reported by several researchers [74,75,76]. However, the development of morphological and anatomical features in response to flooding in most plants is time dependent. Thus, during the initial period of stress, the required energy for survival is mostly generated through anaerobic metabolism [4,63]. 

The internal system of large gas spaces reduces internal volume of respiring tissues and oxygen consumption, thus, enhancing the potential for oxygen reaching the distant underground portions of the plant [17,20,21]. Because of the advantages, the oxygen transport system has been considered as a major mechanism critical to plant’s ability to cope with soil anoxia [17,19,20,21,28,29,30,50,77,78,79,80,81,82]. Diffusion, while the major pathway of root aeration in wetland plants, is not the only means, as rhizome ventilation due to pressurized throughflows of gases has been demonstrated for some species [20,21,83,84]. 

The effectiveness of the gas transport is primarily dependent on two factors: (1) the resistance to diffusion that is proportional to root length and inversely proportional to root porosity; and (2) the oxygen demand along the diffusion path resulting from respiratory needs as well as oxygen leakage from the roots into the rhizosphere [49,85]. Indeed, oxygen demands of roots and rhizosphere, which may include large communities of facultative anaerobes, are competitive for oxygen molecules because in reduced soils these systems compete for the plant pool of oxygen simultaneously [19,79,84]. As soil reduction continues, there is a progressively greater demand imposed upon roots for oxygen [7]. 

It is known that Eh conditions and microbial oxygen demand in the soil are among major factors affecting root oxygen release to the rhizosphere [26]. However, literature concerning the relationship between functional aspects of gas transport within plants and soil Eh conditions is limited. In a few studies that evaluated the relationship, it is evident that intense soil reduction promotes oxygen loss from root to the rhizosphere. For instance, high correlation was found between radial oxygen loss rates from roots and soil Eh intensity; there was an increasingly higher oxygen loss rates as soil Eh became more reduced [14]. However, the enhancement may require soil Eh below some threshold levels [86]. It is reported that in *Spartina patens*, a dominant US Gulf coastal brackish marsh species, root porosity increased as sediment Eh decreased, leading to root porosity of 22% in plants grown at +200 mV while porosity was 45% in plants grown at −300 mV Eh. Radial oxygen loss was significantly greater for plants grown in −300 mV Eh compared to plants grown at +200 mV Eh [87]. Other studies have shown similar patterns of responses for root porosity-soil Eh intensity relationship in wetland plants including swamp and bottomland woody species [17,88]. In contrast, Brix and Sorrell [89] reported that root porosity in two wetland species, *Phalaris arundinacea* and *Glyceria maxima*, did not change in response to a short-term (7–12 days) reducing Eh in root medium. 

Despite the reported increase in aerenchyma tissue formation (and hence porosity) in wetland species in response to reducing Eh conditions, this enhancement may not be sufficient to satisfy the root respiratory needs for oxygen due to the greater radial oxygen loss rates in response to high intensity of reduction. Pezeshki *et al*. [90,91] concluded that despite a substantial enhancement of aerenchyma tissue formation in *Spartina patens*, alcohol dehydrogenase (ADH) activity continued to be higher in flooded than control plants indicating continued oxygen stress in the roots of flooded plants. 

In addition to the effects of the intensity of reduction, differences in Eh capacity among wetland soils may influence many plant functions including oxygen transport, rhizosphere oxygenation, and, thus, many aspects of plant functioning [13,14]. Studies showed that increased Eh capacity under a constant Eh intensity did not have any significant effect on root porosity in *S. patens* but oxygen release from roots to the rhizosphere was increased in response to the increasing Eh capacity [14]. However, the authors reported that there was a threshold Eh capacity beyond which oxygen release remained constant and/or decreased in this species. The response was attributed to the potential effects of several factors such as soil phytotoxins as well as stomatal closure. Nonetheless, the reason(s) for such a response remains unknown. 

## 8. Plant Nutrition

Plant nutrition is influenced by soil flooding and the processes associated with reducing soil Eh conditions. Many factors including soil physicochemical characteristics, nutrient pools, plant developmental and physiological status, and flood-tolerance capabilities are important [22,78,92]. Despite the adaptations reported for wetland plants, various nutritional deficiencies and toxicities may occur. Reduced soil conditions may lead to inhibition of nutrient uptake and transport due to root dysfunction, death [22,61,93,94] and blockages in the vascular and aerenchyma systems resulting from phytotoxin damages [20]. Oxygen supplies to the roots are critical for nutrient uptake and ion transport. Flood-induced stress include reduced water uptake due to root dysfunction that results from soil O2-deficiency including altered cation and anion concentration in xylem sap as well as decreased hydraulic conductance of roots has been reported in the literature for many species [95,96]. The generation of adequate ATP is an important requirement for active uptake that occur via the H+ translocating ATPase in the plasma membrane [43]. Jackson *et al*. [96] reported that flooding degraded root integrity within 10 hours of initiating treatment in tomato plants and solute uptake was deregulated. DeLaune *et al*. [93] using add labeled N-15 measured uptake in cherrybark oak (*Quercus* *falcata* var. *pagodaefolia*) and overcup oak (*Q.* *lyrata*) and reported decreased N-15 uptake under moderate reducing soil conditions as compared to oxidized conditons

There are ample reports of reduction of water and nutrient uptake, disturbance of hormonal balance such as a decrease in gibberellin and cytokinin and an increase in abscisic acid and ethylene in response to flooding [71,72,73]. Under reduced soil conditions, some wetland plants may continue ion uptake partly because of the internal O_2_ supply system but partial anoxia in roots can reduce solute intake [43,61,97]. Nutrient concentrations at toxic levels may be accumulated in tissues under reduced conditions due to higher availability of certain nutrients and root dysfunction [9,98,99]. During prolonged flooding, as soil Eh reduction continues, pH decreases while zinc availability increases leading to high tissue zinc concentrations [100] and reduced ferric and manganic forms that are soluble [6]. Thus, tissue Mn and Fe concentrations are greater than found in plants under aerated conditions [101,102,103]. Leaf discoloration (bronzing) due to high soluble ferrous iron has been reported in some species [49]. 

**Figure 3:**
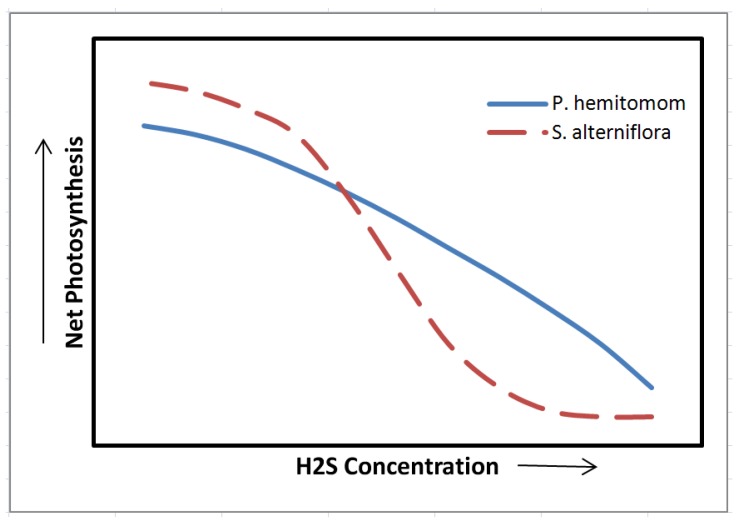
The relationship between net photosynthesis and H_2_S concentration in the sediment for two wetland species, *Panicum hemitomom* and *Spartina alterniflora* (from Pezeshki *et al*. [99,107].

Further soil Eh reduction leads to reduction of sulfate to sulfide by anaerobic microorganisms [104]. Although sulfide is phytotoxic, in most cases wetland plants have the capability to oxidize sulfide in the rhizosphere thus, avoiding or minimizing injury [49]. However, there are several reports confirming that excess soil sulfide may inhibit plant growth [20,21,46,47,48]. The soluble sulfide species including H_2_S are toxic to the roots [49,50]. For instance, sulfate uptake, translocation and accumulation in foliage is documented for several species [105,106]. The inhibitory effect of H_2_S on cytochrome oxidase is disruptive for aerobic respiration and excess cytosolic Fe and Mn is harmful to enzymatic structures [33]. The inhibitory effects of elevated sulfide concentrations on leaf photosynthetic capacity have been demonstrated in several wetland species including *Panicum* *hemitomom* and *Spartina alterniflora* (Figure 3, Pezeshki *et al*. [99,107]. Such photosynthetic response has been attributed to disruption of light reactions [108] and/or photophosphorylation [109], and alterations in activity of photosynthetic enzymes [110,111,112]. Sulfide utilization and injuries have been reported in hypoxic roots and rhizomes of *Phragmites australis* [113]. Sulfide has been implicated as a factor responsible for decreased plant growth and productivity in several wetland species [20,21,99,114]. 

## 9. Plant Water Relations and Gas Exchange

Factors associated with low soil Eh conditions can influence plant water relations through stomatal closure and slower water uptake than under aerated conditions [23,95,96,115,116,117,118]. Increased internal water stress and leaf dehydration leading to stomatal closure have been reported in some species due to a decrease in root permeability when roots were flooded [23,78,119]. Reduced water uptake due to root dysfunction may include altered cation and anion concentration in xylem sap. Decreased hydraulic conductance of roots has been reported in the literature for many species [95,96]. 

The extent of development of internal water stress reported for some species shows a wide range, however, in most cases the initial stomatal closure occurs in the absence of significant changes in plant water status [120,121,122]. The rapid stomatal closure and maintenance of a favorable water status thus, is likely due to low transpiration rates for which a slow water absorption rate by roots may sufficiently compensate rather than a sustained root conductivity [123,124]. Else *et al*. [116] found that in some species, flooded roots led to rapid stomatal closure via a root-shoot signaling mechanism, thus avoiding the possibility of dehydration. Many wetland species initially close stomata in response to soil flooding, however, exceptions have been reported in the literature. For instance, stomata in certain mangroves did not respond to Eh as low as −180 mV over short-term experimental exposures [125,126]. The stomatal closure is concomitant with a reduction in photosynthesis. However, under prolonged soil reduction stomatal reopening may occur leading to photosynthetic recovery [127]. The degree of resumption of stomatal functioning appears to be dependent on species, duration of reducing conditions, and the intensity of soil reduction. 

**Figure 4:**
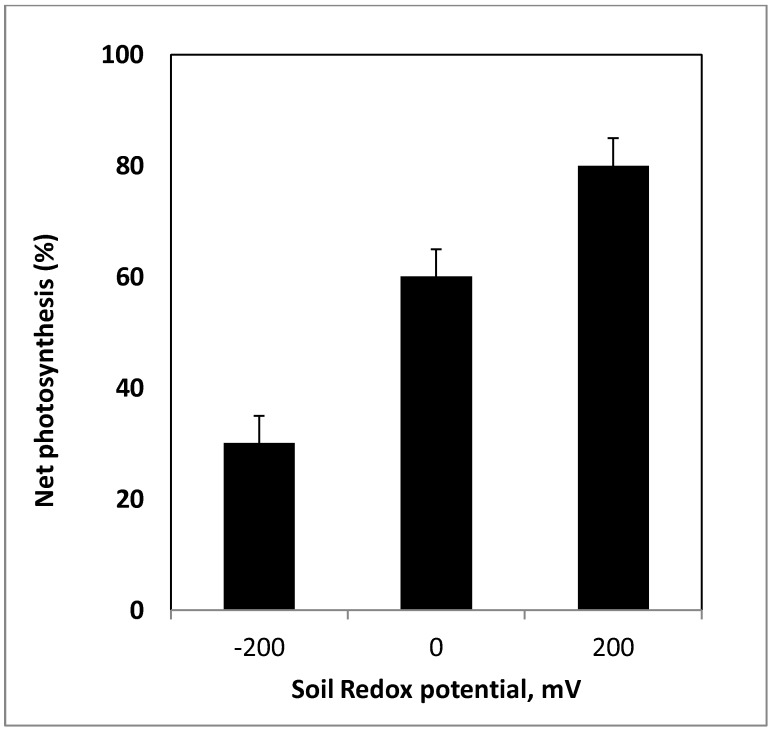
Changes in net photosynthesis of *Typha domingensis* in response to soil redox conditions. Values are presented as percent of controls (from Pezeshki *et al*. [157]).

**Figure 5:**
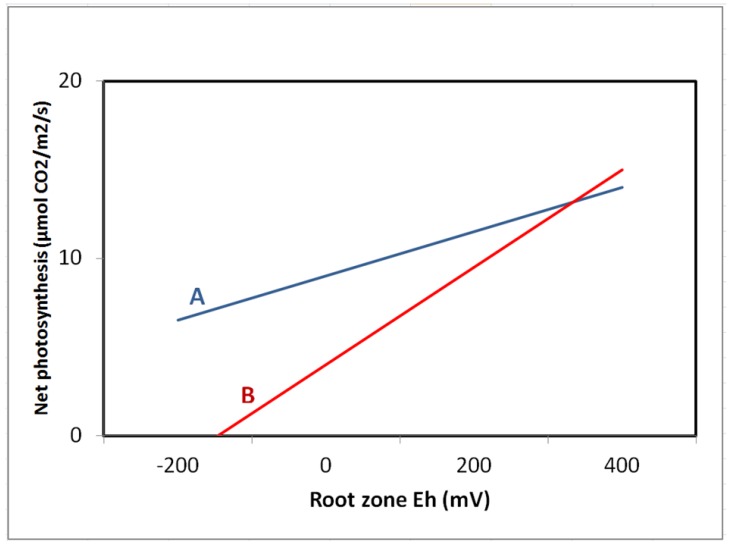
Response of light saturated leaf photosynthesis to root-zone redox potential (Eh) for 5-year-old (A) *Fraxinus pennsylvanica* and (B) *Quercus bicolor* in a created wetland (from Pennington and Walters [28]).

**Figure 6:**
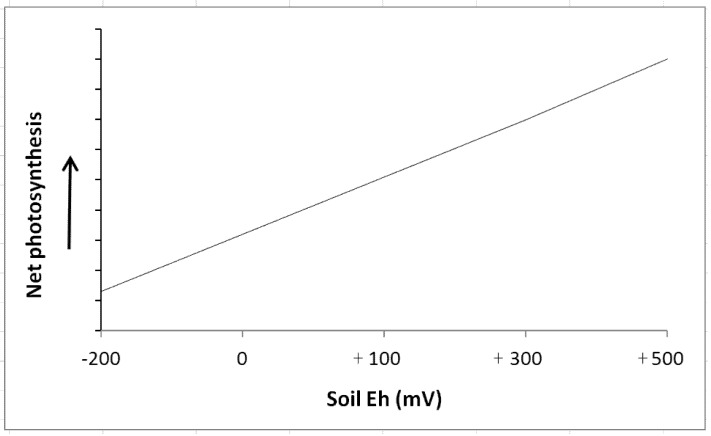
A generalized relationship between photosynthetic activity in wetland plants and soil redox potential (from Pezeshki [22,23]).

Decreases in photosynthetic rates have been reported for some species in response to reducing soil Eh conditions; Figure 4, Figure 5 [23,28,81,82,117,128]. For example, DeLaune *et al*. [7] studied photosynthetic responses to soil Eh using Titanium-citrate solution. The authors noted that the intensity of reduction in growth medium had significant influence on plant phtosynthetic activity in marsh grass *Spartina patens* as Eh below −200 mV led to decreased photosynthesis. Krauss *et al*. [128] noted decreased stomatal conductance and net photosynthesis for baldcypress (*Taxodium* *distichum*) plants flooded in a greenhouse. As soil Eh dropped to −40 to −70 mV range, stomatal conductance reduced 36% and net photosynthesis dropped 40% compared to controls. In general, reducing soil Eh conditions adversely impact photosynthetic rates in wetland plants (Figure 6) although the extent of the impact varies across species as shown in Table 1. Additional studies by Pennington and Walters [28], Gravatt and Kirby [129], and Krauss *et al*. [128] have further confirmed the inhibitory effects of low soil Eh on photosynthesis of woody species. The photosynthetic decline may be attributed to a combination of diffusional limitations on gas exchange due to stomatal closure and metabolic (non-stomatal) inhibition [127]. However, the contribution of each component requires further assessment. In response to low soil Eh in range of −200 to +100 mV, a shift in light response curves was noted in some woody species suggesting the adverse effects of low Eh on the photosynthetic capacity of the leaves [88]. 

**Table 1:** Photosynthetic responses of selected marsh plants and wetland/bottomland woody species to the intensity of soil reduction. Values represent percent reduction in net photosynthetic rates compared to plants grown under oxidized (Eh > +400 mV) conditions or percent of pre-stress levels.

Species		Soil Eh (mV)	Photosyn. Response (%)	Reference
Marsh plants				
	*Typha domingensis*	+200	18	[157]
		0	49	[157]
		−200	75	[157]
	*Cladium Jamaicense*	+200	24	[157]
		0	46	[157]
		−200	100	[157]
	Spartina alterniflora	<−200	15–21	[131]
	*Spartina patens*	+230	7	[91]
		−110	18	[91]
Woody speccies				
	*Quercus lyrata*	+340	54	[93]
		+175	66	[93]
	*Ligustrum sinese*	+200/−200	86	[132]
	*Taxodium distichum*	−70	5	[158]
		−160	22	[158]
	*Taxodium distichum*	+350/+175	5	[159]
	*Taxodium distichum*	−40/−70	36	[128]
		+18/+172	Decreased/recovered	[160]
	*Salix nigra*	−130	53	[158]
		+50 to −80	5	[86]
	*Quercus nuttallii*	+100/−220	35–68	[88]
	*Quercus nuttallii*	+350/+175	21	[159]
	*Quercus falcata*	+100/−220	65–87	[88]
	Quercus michauxii	+350/+175	58	[159]

Chlorophyll fluorescence has been used to assess intact plants for detecting stress effects such as flooding effects [130] particularly the effects on PSII functioning. Utilizing this technique, Pociecha *et al*. [117] reported that in flooded *Vicia faba* L. plants, there was an apparent damage to photosynthetic apparatus as indicated by lower Fv/Fm compared to control plants. However, in wetland plants such as *Salix nigra*, Eh drop to the range of −50 to −100 mV did not have any significant effect on Fv/Fm [86]. This Eh range represents a moderately reduced soil conditions thus may explain the lack of PSII response in a wetland species such as *S. nigra*. Additional research is needed to assess PSII response to low soil Eh in wetland plants. 

Photosynthetic recovery is reported for some wetland species despite the continuation of low soil Eh conditions provided that the soil is moderately reduced [88,127,131,132]. Soil Eh below +150 mV led to decreased net photosynthesis in *Alnus japonica* seedlings and the rate of recovery was correlated with the development of adventitious roots [81,82]. Even if a substantial recovery of photosynthetic rates occur, the impact of initial reduction and the delayed and/or slow recovery on plant survival and growth may be substantial due to the disruption of carbon fixation and decrease in photosynthate production [28,61,88,128,129]. 

What are the mechanisms through which the low soil Eh condition reduces leaf photosynthetic activity in many wetland plants? There are numerous factors that may be responsible for the decrease of net photosynthesis. For instance, ethylene accumulation has been implicated and the effects may be due to loss of photosynthetic capacity of mesophyll [133]. Low soil Eh conditions could lead to reductions of net photosynthesis due to decreased leaf water potential, reduced rubisco activity [66], disruption in photosynthate transport [61], alterations in source-sink relationship or reduced sink demand [23,33,134,135]. Other factors that may contribute to the reduction in photosynthetic capacity include low leaf chlorophyll content and/or leaf chlorophyll degeneration, and dysfunctioning of PSII. Recent reports showed that flooding led to limitations on the quantum efficiency of PSII in certain crop plants [116,136]. Leaf chlorophyll content decreases in some wetland plants that are subjected to reduced soil conditions [137]. 

Rubisco is an important enzyme in photosynthesis that catalyzes carboxylation yielding two molecules of 3-phosphoglycerate and oxygenation that produces one molecule of 3-phosphoglycerate and one molecule of 2-phosphoglycolate [138]. Because activity of this enzyme is critical for photosynthesis, flood-induced reductions of photosynthesis may be due, at least partially, to decreases in rubisco activity. In addition, the recovery of rubisco activity may contribute to the observed recovery of net photosynthesis in some wetland plants [22,66,127]. Indeed changes in rubisco (EC 4.1.1.39) activity may be an early signal contributing to the reduction of leaf photosynthesis in flooded plants as reported in the literature [66,139,140]. In some crop species, stress leads to reduced rubisco activity and enhanced rubisco degradation [65,141,142]. Li *et al*. [143] showed that decreased gene expression of oxygen evolving complex, large subunits of Rubisco and ferredoxin in response to flooding may have contributed to the observed photosynthetic reduction.

Low soil Eh conditions influences translocation of various photosynthetic products as reported for some [61,144,145]. The effects include low ATP production resulting from disruption of the oxidative phosphorylation [56,146,147], carbohydrate synthesis, transport, allocation, and utilization [78,148,149,150]. Carbohydrate allocation patterns and translocation rates appear to be critical for withstanding hypoxic condition [149,150,151]. However, the specifics of wetland plant responses remain to be evaluated. 

## 10. Growth and Biomass Production

**Figure 7:**
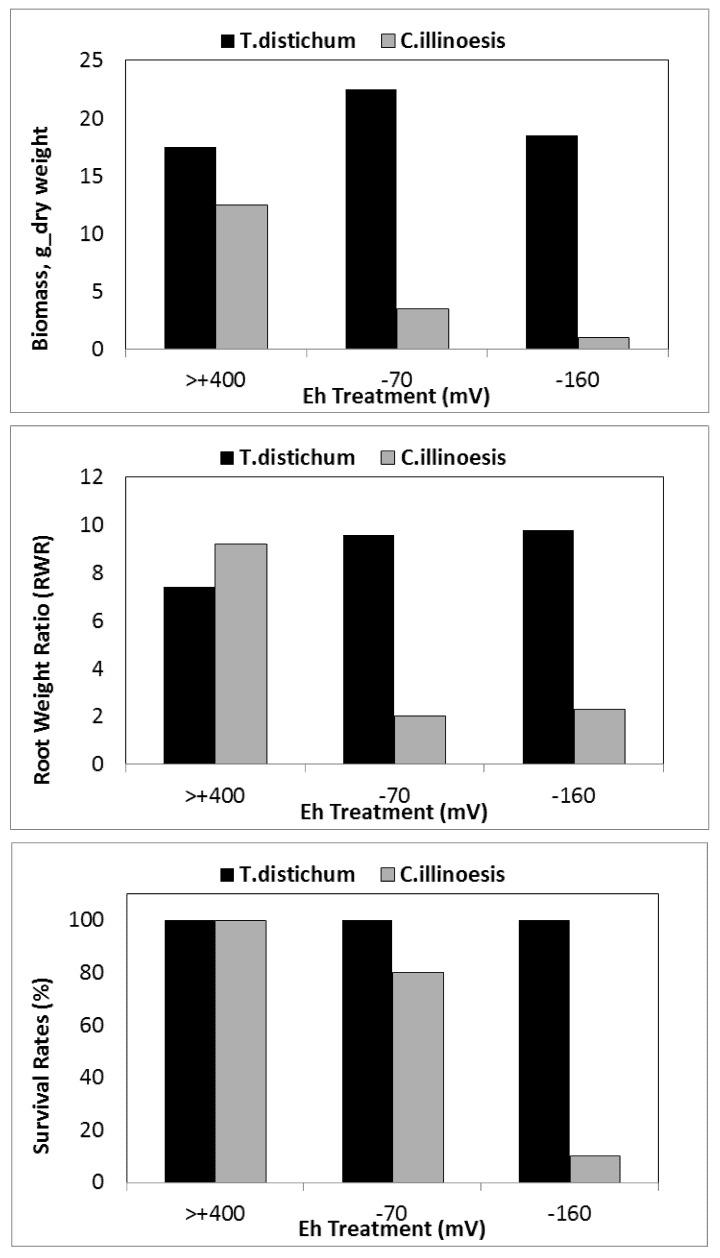
Biomass accumulation, root/weight ratio, and survival of two woody species *Taxodium distichum* and *Carya illinoesis* in response to changes in soil Eh conditions (from Pezeshki and Delaune [158]).

**Figure 8:**
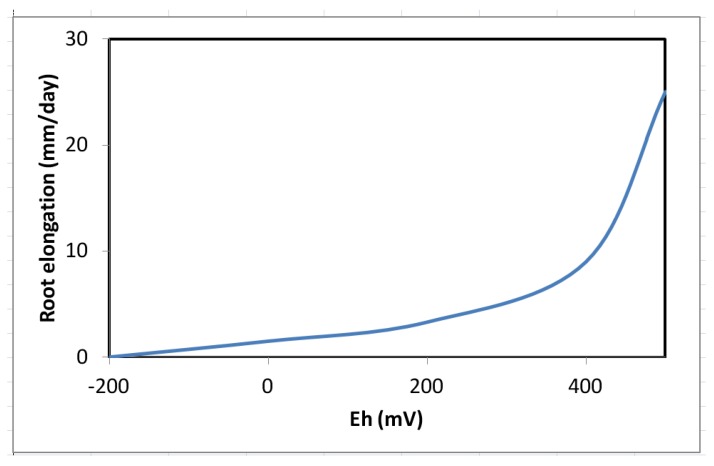
Responses of root elongation in *Spartina patens*, a brackish marsh species, to soil redox potential (Eh) (from Pezeshki and DeLaune [153]).

Decrease in survival and biomass accumulation in response to low soil Eh conditions is reported in certain wetland species. For example, while *T. distichum* remained relatively unaffected by Eh as low as −160 mV (Figure 7a–c), *Carya illinoesis* showed dramatic response at Eh around −70 mV (Kludze and DeLaune [13,14,23,61,86,87]. Significant changes are also reported in root to shoot ratio in some species as the effects of soil reduction are usually more drastic on root systems than shoots [86,114,152]. While a range of responses to low soil Eh conditions may occur in a given species, these changes, nevertheless, occur within the context of an overall reduction in biomass accumulation as reported for flooded plants of many species (Pezeshki [23] and the references cited therein). Root and shoot dry weights in *S. patens* decreased by 40% and 25% as soil Eh dropped from +200 mV to −300 mV, respectively. Results clearly indicated the influence of soil Eh intensity on growth of this dominant U.S. Gulf coast marsh species. It also was demonstrated that roots were more sensitive to Eh intensity than shoots (also data by Kludze and DeLaune [87]). In general, Eh reduction has pronounced effects on root elongation (Figure 8). Similar conclusions were drawn from a study on a flood-tolerant woody species *Taxodium distichum* [17]. Pezeshki and DeLaune [153] reported cessation of root growth in *S. patens* at soil Eh of −100 mV. In addition, Pezeshki *et al*. [90] noted smaller root system in *S. patens* under reducing soil conditions and concluded that such reduction in sink size, may in part, be responsible for a negative feed-back inhibition of photosynthesis thus, reduction in productivity of this species.

The increase in oxygen loss rates reported under reducing soil Eh [14,87] may explain the reductions in root growth of several wetland species under low soil Eh conditions. *Cladium jamaicense* and *Typha domingensis* produced significantly less biomass at Eh of −150 mV compared to plants grown at oxidized Eh of +600 mV [155]. Inhibitory effects of low soil Eh on photosynthesis and growth of woody species has also been reported by Pennington and Walters [28]. Root growth is an energy dependent process requiring oxygen, thus, under flooded conditions, root functioning is affected rapidly because molecular oxygen is required as an electron acceptor for oxidative phosphorylation [33,154]. Root elongation was inhibited in some bottomland woody species when soil Eh fell below +350 mV [152]. Interestingly, the +350 mV is the approximate Eh level that signifies the onset of oxygen disappearance from the soil system [7]. Root penetration depth was also adversely affected under low soil Eh treatment resulting in development of shallow root system different in architecture than those of plants growing in aerated (Eh > +400 mV) conditions [152,153]. The critical threshold Eh that inhibited root elongation differed among wetland species ranging from +300 mV to −200 mV [152,156].

**Figure 9:**
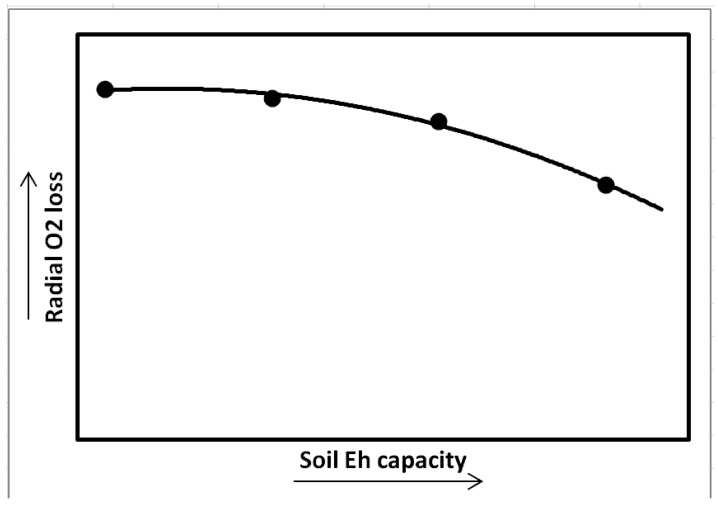
Radial O_2_ loss (ROL) in *Spartina patens* in response to changes in soil reduction capacity while soil reduction intensity was maintained at −200 mV (Kludze and DeLaune [14]).

Soil Eh capacity also influences wetland plant gas exchange and growth [13,14]. In the study, plants were grown under controlled Eh to examine the effect of Eh on CO_2_ fixation. Soil redox capacity was imposed by application of different levels of extra energy source, by adding granular D-glucose to the growth medium, while maintaining Eh intensity at −200 mV. Increases in soil Eh capacity led to decreased photosynthesis, root growth, and biomass in *Oryza sativa* while enhanced root porosity and radial oxygen loss rate (Figure 9). Other studies demonstrated that plant carbon fixation, root, and shoot growth were significantly inhibited in *S. patens* under increasing soil reduction capacity. Root and shoot dry weights decreased by 70 and 37% in high reduction capacity conditions compared to control plants, respectively [14]. 

## 11. Concluding Remarks

There is a sufficient body of data to show that many plant functions are influenced by low soil Eh conditions. Wetland plant response is dependent on several factors including the species, duration of soil reduction, the timing, the intensity, and the capacity of soil reduction. The reducing condition of the soil is a major factor in wetland ecosystems that influences plant survival, growth, and productivity. Thus, quantifying soil reduction is critical to the understanding and interpretation of wetland plant responses to such conditions. In addition, plant response to low soil Eh conditions also reflects species’ ability to respond to such conditions by utilizing a variety of morphological, anatomical and metabolic defense mechanisms. Nevertheless, many wetland species including those that possess a wide range of tolerance/avoidance capabilities to cope with low soil redox conditions are impacted negatively. The impact is a reflection of the fact that reducing soil conditions encompass not only soil oxygen deprivation but also production of various compounds in the soil, many of which considered highly phytotoxic. Thus, soil reducing conditions exert substantial influence on critical plant processes including gas exchange, water relations, photosynthate partitioning, translocation, hormonal balance, nutrition, growth, and biomass production. 

Based on the limited available data, both intensity and capacity of reduction appear to influence plant functioning in wetland ecosystems although the roles of both factors need further investigations. In wetland soils, plants are faced with a substantial demand for oxygen from roots and soil microbial populations, the potential for loss of oxygen to soil through root radial oxygen loss that could improve bulk soil Eh conditions but nevertheless may impose plant internal O_2_ deficiency, and the adverse effects of soil phytotoxins that are by-products of soil reduction. The severity of oxygen loss and the effects of reduction intensity and capacity on plant functioning are clearly common but vary across species. The need for additional data on various aspects of plant functioning and growth in wetland ecosystems in response to soil redox conditions, in terms of the intensity and the capacity, as well as the specific effects of soil phytotoxins is clear. 
